# EMu: probabilistic inference of mutational processes and their localization in the cancer genome

**DOI:** 10.1186/gb-2013-14-4-r39

**Published:** 2013-04-29

**Authors:** Andrej Fischer, Christopher JR Illingworth, Peter J Campbell, Ville Mustonen

**Affiliations:** 1 Wellcome Trust Sanger Institute, Wellcome Trust Genome Campus, CB10 1SA, Hinxton, Cambridge, UK

**Keywords:** cancer genomes, expectation-maximization, chromatin state, breast cancer, mutation clustering

## Abstract

The spectrum of mutations discovered in cancer genomes can be explained by the activity of a few elementary mutational processes. We present a novel probabilistic method, EMu, to infer the mutational signatures of these processes from a collection of sequenced tumors. EMu naturally incorporates the tumor-specific opportunity for different mutation types according to sequence composition. Applying EMu to breast cancer data, we derive detailed maps of the activity of each process, both genome-wide and within specific local regions of the genome. Our work provides new opportunities to study the mutational processes underlying cancer development. EMu is available at http://www.sanger.ac.uk/resources/software/emu/.

## Background

The development and progression of cancer is now widely viewed as an evolutionary process [1-3]. Mutation is a key component of this process: cancer development occurring through the progressive acquisition of driver mutations [4]. Cancer genomes often display large numbers of mutations when sequenced; indeed, whole-genome sequencing studies have identified tens of thousands of mutations within individual tumors [5]. The vast majority of these mutations are believed to be passenger mutations not conferring a growth advantage to the cancer cell [4]. They do, however, carry useful information about the forces to which the cancer genome has been subjected during its evolution.

A variety of physical, chemical and biological processes are known to lead to mutations in cancer. Sequencing of different cancer types has shown strong and distinct biases for particular mutation types in particular cancers, such as a propensity, due to UV radiation [6], for C:G > T:A mutations in melanoma [7], and an increased number of C:G > A:T mutations resulting from chemicals in tobacco smoke [8,9]. Deactivation of mismatch repair mechanisms can also lead to mutational biases [10]. We here consider the task of identifying mutational processes at work during cancer development. We assume that these processes are distinct, in that each process leaves a different characteristic mark on the cancer genome. Given sequence data from multiple tumors, in which mutational processes have been active to different extents, we aim to infer the number of elementary mutational processes, their signatures and the contribution of each process towards the spectrum of mutations observed in each tumor. The task itself is an important one: identifying mutational processes is a step towards understanding the causative mechanisms of cancer. A detailed account of the mutational processes active in a cancer could inform a neutral null model of mutations when inferring signals of selection in cancer genomes. The presence of a mutational process could indicate a driver mutation, for example in a gene controlling DNA repair.

Computational methods addressing this task have been developed [11,12] and, more recently, applied to whole genome sequencing data from multiple breast cancer samples [13]. However, as previously implemented, these methods have significant limitations. Firstly, the opportunity for mutations to occur in a given genetic sequence has not been explicitly accounted for. This is important as the observed outcome of a mutational process depends on the composition of the sequence upon which it acts. For example, a process which produces C > T transitions at CpG sites may act with uniform activity across a genome, but it will be realized and observed more often in regions with a higher density of these dinucleotides. Secondly, while considering mutational processes genome-wide, previous studies have not examined the action of different processes within smaller regions of the genome. Unevenness in mutation rate across a cancer genome has been observed, associated with bias in the action of DNA repair mechanisms [5] and with the distribution of histone modification marks [14], suggesting that mutational processes may act differently in different regions of the genome. Changes in mutational opportunity, noted above, are likely to be particularly important within small regions of the genome. Copy number variation, which can be prevalent in cancer [15], substantially alters the mutational opportunity in regions of ploidy change, while different regions of the human genome may radically differ in their internal sequence composition [16]. Finally, previous methods have not explicitly considered the stochastic manner in which mutations are introduced into the genome. Stochasticity, together with the discrete nature of mutation, leads to specific patterns of noise in the observed mutation spectra. This noise is amplified when smaller subsets of the genome are analyzed, making an explicit probabilistic treatment of the data indispensable.

We here describe a novel method, EMu, based upon the expectation-maximization (EM) algorithm [17], to infer the number of elementary mutational processes and their spectra from cancer sequence data. Use of an explicitly probabilistic framework allows our method to account for biases in the mutational opportunity and to accommodate noisy data. Using simulation results, we show that incorporating the mutational opportunity is critical in identifying the correct number of mutational processes within this model. As a likelihood-based method, the EM approach deals naturally with the stochastic nature of mutational processes, and enables us to use model selection criteria, such as the Bayesian information criterion (BIC) [18], to decide which number of processes has the strongest statistical support. Meaningful error estimates of the inferred mutational signatures can be derived either analytically or numerically with Markov chain Monte Carlo (MCMC) methods. By extending the rationale of the parameter-learning algorithm to smaller subsets of a cancer genome, the local activity of different processes can be measured. Further, our method is computationally efficient, allowing for the rapid analysis of thousands of cancer samples.

We apply our method to the sequences of 21 breast cancers, described in an earlier study [19], and compare the inferred mutational spectra to those previously reported. Next, we examine local variation in the impact of the different mutational processes; we show that regions of *kataegis*, a phenomenon of regional hypermutation identified in breast cancer [13], are dominated by one specific mutational process. Finally, we take advantage of annotation data from the ENCODE project [20] to study variation in mutational processes associated with chromatin state. The ENCODE project provides segmentations of the human genome in different functional chromatin states [21], mainly based on the distribution of histone modification marks. In cancer, a recent study has identified strong correlations of somatic mutations with some of these histone modifications, notably H3K9me3 [14]. We here present statistical evidence that mutational processes act differently in regions of differing chromatin state in breast cancer. In particular, one of the mutational processes, producing mostly C > T mutations at CpG sites, is strongly underrepresented in promoter regions and enhanced in heterochromatin regions. This suggests that this process is correlated with DNA methylation, as would be the case for spontaneous deamination [22].

## Results

### Validation against simulated data

**Figure 1:**
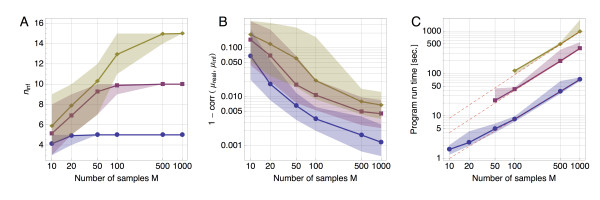
**Performance**. Results of the performance test of the EM method on simulated mutation data. Each simulated tumor belongs to one of up to three different cancer types, where in each type there are five independent processes active. The total number of processes per data set is thus 5 (blue), 10 (red) or 15 (yellow). Here, we show the scaling of different observables as a function of sample size (calculated over 50 replicates for each combination of *M *and *n*). **(A) **The number of processes present in the data is determined via the BIC. Shown are the median (line), the smallest and the largest (shaded area) number of inferred processes. **(B) **The correlation between the real and the inferred mutation spectra (the difference from 1 is plotted). **(C) **The time until completion of the inference program scales approximately linearly with *M *(for constant *n*; the fits above correspond to 0.93, 0.99 and 1.02). (B and C show the median with the 10% and 90% quantiles.). BIC: Bayesian information criterion; EM: expectation-maximization.

We here show the performance of the EM inference method with the help of simulated data. Cancer mutation data were generated for increasing numbers of tumor samples (*M *∈ {10,20,50,100,500,1000}) and different numbers of underlying mutational processes (*n *∈ {5,10,15}), where we tried to replicate the distribution of observed mutations in the breast cancer data set (exact simulation parameters are detailed in Additional file 1). In each case, increasing the sample size improved the ability of the method to distinguish between mutation processes; five mutational processes could be distinguished from twenty tumor samples in the vast majority of cases (Figure 1A). But even where not all processes were found, for those that were identified the mutational spectra showed a strong correlation with their real counterparts (Figure 1B). Using BIC for model selection, the number of processes was never overestimated. However, when trying to perform the inference, ignoring the mutational opportunity led to consistent and significant overestimation of the number of processes as given by BIC (see Additional file 1).

Our implementation of the EM method (described further in Materials and methods) led to rapid inference of mutational processes and their respective activities; the mean convergence time scaled close to linearly, or better, with the number of samples (the increased evidence provided by a greater number of samples can lead to faster convergence). As such, calculations involving thousands of samples could feasibly be conducted on a standard computer (Figure 1C).

### Mutational processes in breast cancer

**Figure 2:**
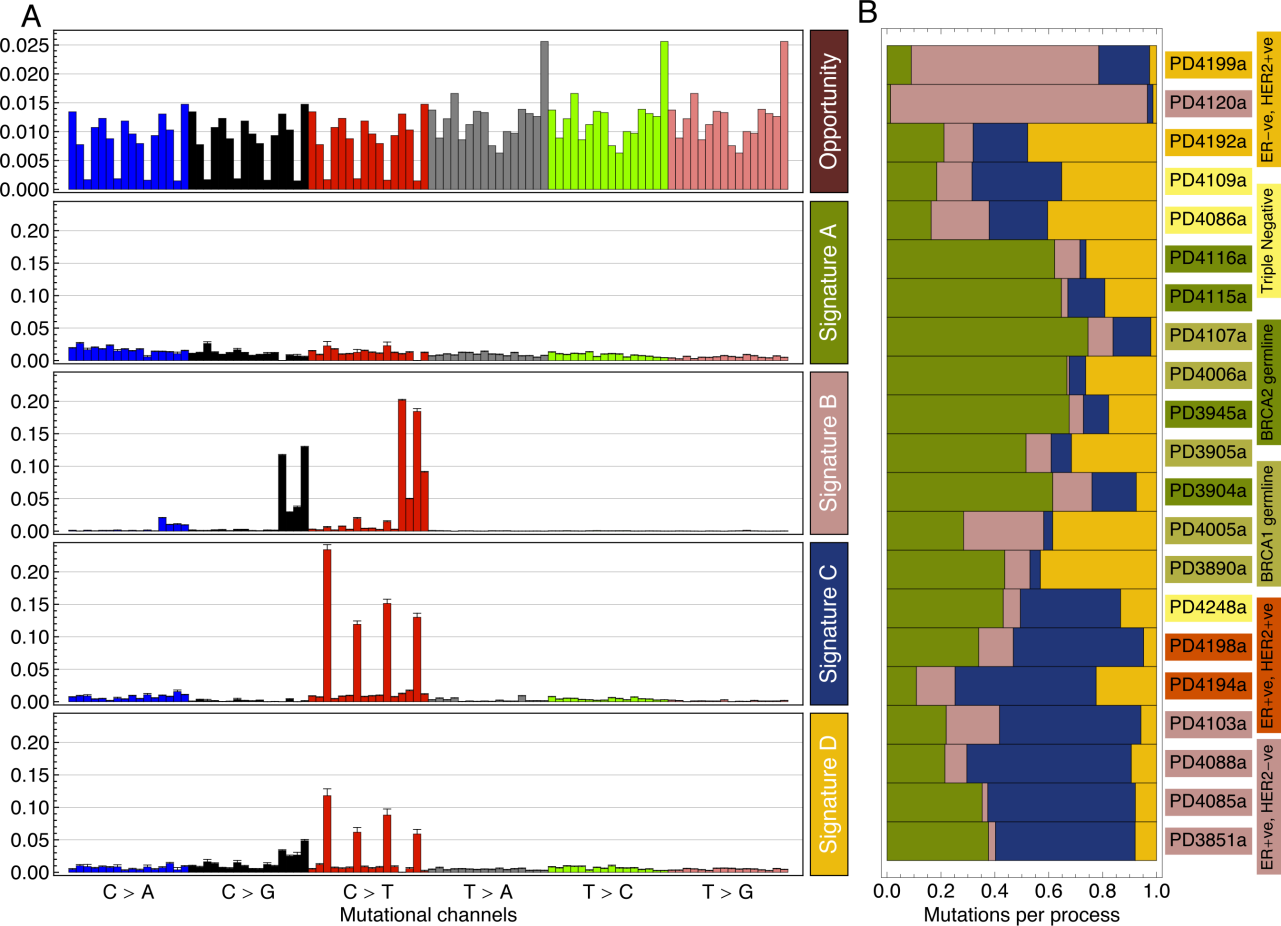
**Mutational spectra and tumor composition**. **(A) **The mutational opportunity spectrum of the human genome across 96 trinucleotide channels and the mutational signatures found in the breast cancer data. For each mutation, the 16 sequence contexts are ordered by the 5' and then the 3' base in the order A, C, G and T, that is, from ApCpA, ApCpC to TpCpG, TpCpT. The error bars were estimated analytically (see Additional file 1). **(B) **The contributions of each mutational process towards the mutations for each tumor. The correlation with cancer subtype matches that reported in [13].

A recent study [13] has identified independent mutational processes acting in 21 breast cancers. Applying our probabilistic inference method to this particular data set, we identified four mutational processes (Figure 2A). Judging from the simulated data (Figure 1), 21 independent samples would likely be sufficient to identify five spectra (depending on their similarity and noise level), suggesting our inference to be robust. The four spectra identified by the EM method have very pronounced features, such as the bias for C > T mutations at CpG dinucleotides in processes C and D. Process B shows a bias for C > G and C > T mutations in TpCpX contexts, whereas process A seems to be a random background process.

Across the human genome, the mutational opportunity varies between channels by as much as 17-fold, making this an important factor in the derivation of mutational spectra. In the breast cancer data set, this is compounded with a prevalence of large-scale copy number changes [13,19]. This results in a mutational opportunity spectrum that is highly tumor specific.

The global activities of each of the mutational processes within each cancer showed clear differences between the cancer samples (Figure 2B). For example, mutations in tumor sample PD4120a almost exclusively resulted from process B. PD4120a is an outlier in the total number of mutations, such that it has a large influence on the compositions of the identified spectra. However, our inference proved robust to this; re-running the analysis with this tumor excluded resulted in spectra that were very similar to those found in the complete data scenario (see Additional file 1).

### Local analysis reveals regions strongly targeted by individual processes

**Figure 3:**
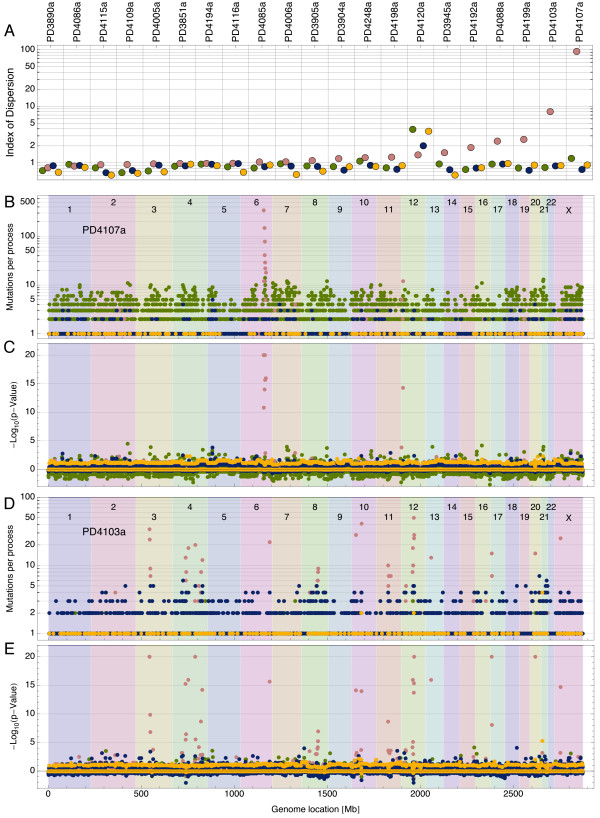
**Clustering of mutations within the cancer genome**. **(A) **The index of dispersion (corrected for the heterogeneity in spectrum-opportunity overlap and ordered by process B value) for all 21 tumors and the elementary processes (logarithmic scale). Process B shows a tendency to be over-dispersed in some tumors (values larger than 1) indicating local clustering of mutations in the corresponding cancer genomes. **(B) **Local assignment of mutations to processes for sample PD4107a by chromosome. **(C) **The corresponding *P *value with respect to the null hypothesis that the mutations are randomly distributed in the genome (positive values signify a surplus of mutation, negative values a deficit; values are capped at 20) by chromosome. The *kataegis *events in chromosomes 6 and 12 can clearly be attributed to process B. **(D) **and **(E) **show the same information for sample PD4103a, which harbors many more *kataegis*-like events, albeit of different magnitude. Green: process A; pink: process B; blue: process C; yellow: process D.

To localize the regions of the genome where the different mutational processes are active, we used the globally inferred mutational signatures to estimate the activity of each process for each sequence window of 1 Mb (further explained in Materials and methods). Variation in the local activity of individual mutational processes was seen across each cancer genome, especially in process B. To gain a first overview of the extent of mutational heterogeneity, we measured for each process in each cancer sample an index of dispersion (the ratio of the variance to the mean of mutations per window, see Additional file 1), corrected for mutational opportunity, where a value of 1 corresponds to an unbiased and homogeneous distribution of mutations across the genome. Substantial over-dispersion was identified in process B, most obviously in tumor sample PD4107a, which shows an index of dispersion close to 100 (see Figure 3A).

Clustering of large numbers of mutations within a small region of the genome, termed *kataegis*, has previously been identified in the breast cancer genomes [13] considered here, being found in 13 of the 21 tumors. In previous work, *kataegis *events were found to largely comprise C > T and C > G mutations. We now see that *kataegis *is strongly associated with mutational signature B.

A higher resolution picture of mutational variation identified regions of significant mutational clustering. For each 1 Mb region of the genome, the number of mutations identified from each process was evaluated against the null hypothesis that each process distributes its mutations randomly in the cancer genome, according to a globally uniform activity, modulated only by local opportunity. Inspection of the *P *value as a function of genome location in tumor sample PD4107a highlighted very clearly the previously reported *kataegis *event in chromosome 6, but in addition, found a smaller, but highly significant, event at the beginning of chromosome 12 (see Figure 3C).

### Mutational processes and chromatin state

**Figure 4:**
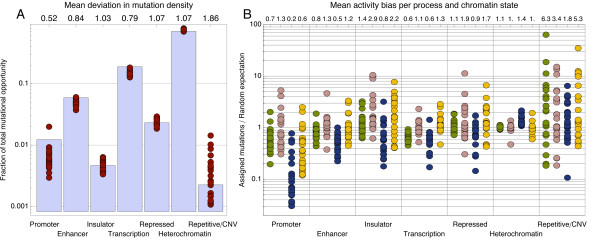
**Mutational processes and chromatin state**. **(A) **Comparison of the fraction of mutational opportunity in each chromatin state portion of the human genome to the fraction of actual somatic mutations found in them (red dots). The numbers at the top show the mean over- or under-representation of somatic mutations per chromatin state. **(B) **Of the mutational processes identified in breast cancer, process C shows the strongest variation with chromatin state. In promoter regions, process C mutations are suppressed by 80% on average, whereas in heterochromatin regions there is, on average, a 40% surplus of them. Green: process A; pink: process B; blue: process C; yellow: process D.

The localized analysis can be carried out for any well-defined subsets of the genome. The segmentation of the genome into functionally relevant chromatin states by the ENCODE project [20,21] is of particular interest. Indeed, the regional density of mutations was found to vary by the chromatin state in human mammary epithelial cells (HMECs) (Figure 4A). Comparing the fraction of mutational opportunity in each chromatin state to the fraction of actual mutations observed showed there to be 7% more mutations in heterochromatin segments than would be expected by chance, but almost 50% fewer mutations in promoter regions. Using our localized inference method we investigated the sensitivity of the mutational processes to chromatin state. To this end, we measured the extent to which the local activity of a mutational process depended upon the chromatin state of a genomic segment (see Additional file 1). The mutational process C (C > T at XpCpG sites) shows a highly significant change in activity in both heterochromatin and promoter regions. This signal is consistently observed in all 21 cancers (see Figure S5 in Additional file 1). To quantify the size of this effect, we compared the number of mutations that were assigned to a specific process in each chromatin state to the number that would be expected if the process acted uniformly across the genome. In Figure 4B, we show the ratio of these two numbers for each process in each chromatin state and for each of the 21 tumors. In heterochromatin regions, the activity of process C is enhanced in all tumors, on average by 40%, a large effect given the size of that genomic segment. In promoter regions, process C is consistently suppressed, on average by 80%. This observation raises the possibility that process C is dependent upon DNA methylation state. Process C consists largely of C > T mutations at CpG sites. Such mutations have been observed to occur more frequently at CpG sites when the cytosine is methylated, as a result of spontaneous deamination [22,23]. In promoter regions of active genes, there are more CpG sites than usual (increasing the opportunity for process C), but predominantly they are unmethylated (reducing the observed activity of process C).

## Discussion

The main import of this study is the demonstration of a robust and flexible probabilistic method to extract the elementary mutational processes and their signatures from large-scale cancer mutation data and to localize process activity within the cancer genomes. As an application of the EM paradigm, this method is solidly grounded in information theory and thus allows for a robust separation of the signal from the noise in the data. Using extensive simulated data sets, we have shown that it is highly scalable and can be conveniently used to analyze hundreds to thousands of cancer genomes.

In the second part of this study, we have demonstrated how one can infer where the different mutational processes are localized within individual genomes. With respect to breast cancer, we have made some unexpected observations: first, process B is most prevalent in the mutator-phenotype tumor PD4120a, but is also strongly associated with the phenomenon of *kataegis*, that is, mutational thunderstorms. Second, comparing the process-specific distribution of mutations in the genome to its chromatin state, process C seems to be strongly suppressed in promoter regions and significantly enhanced in heterochromatin regions. The chromatin state annotation that was used here was derived from experiments performed upon healthy tissue. An interesting extension of this work would be to study the correlation between the mutational processes and the functional and epigenetic annotation of the actual cancer genomes that they were found in. This kind of data is as yet unavailable but certainly within reach of current technologies.

The inference method presented in this study is not limited to the 96 trinucleotide mutation channels. Additionally, one could include such information as the strandedness of mutations or whether a mutation was an early or late event in the life history of the cancer [19]. This could, potentially, shed light on the temporal sequence with which different mutational processes assault the cancer genome. For all such extensions it has to be ensured that the mutational opportunity is taken into account appropriately. Due to chromosomal rearrangements and large-scale copy number changes during somatic evolution, this mutational opportunity is actually a dynamic quantity [19]. As an approximation, we used the copy number state of the cancer genomes at the time of sequencing to calculate mutational opportunity. For example, a duplication event on one parental copy of a chromosome might have happened in the late stage of a cancer's evolution. The mutational opportunity for this duplicated segment would thus be increased by 50% (from two to three copies) for the remaining time until sequencing. In this work, we have assumed that this increased mutational opportunity was available to all the mutational processes throughout. With more detailed information about the relative timing of copy number changes one could potentially construct an improved effective mutational opportunity.

The inference method that we propose is easily generalized to mutation data found in subsets of the cancer genome, for example from exome sequencing studies, as long as the correct mutational opportunity is used. In its present form, the inference of mutational signatures does not explicitly account for any effects of selection on the outcome; we implicitly assume that all mutations are in effect neutral with respect to cancer evolution. However, the signatures we infer here might be of help in a future search for signals of selection in cancer genomes. Any claim for the presence of selection in a sequence must be contrasted against the predictions of a null model of neutral mutations, of which the present mutational signatures could form the basis. To further emphasize the importance of understanding quantitatively the mutational processes active in cancer, we note that passenger mutations themselves have been recently proposed as a potential therapeutic target [24].

Potential future applications of this method include the cataloguing of mutational signatures across different cancer types, identifying shared and cancer-type-specific mutational processes (and, hence, a better classification of cancers by their process composition) and finally, a more refined, cancer-type-dependent null model to identify causal cancer driver variants via signals of selection.

## Conclusions

We here present a probabilistic model for cancer mutations and a robust method to identify the mutational signatures of processes that were active in sequenced cancers. Under explicit consideration of the opportunity for different mutations to occur, we infer the localization of the mutational processes in individual cancer genomes. Using recent data on breast cancer tumors, we have demonstrated how a comparison of the distribution of mutational processes with functional annotation of the genome can lead to new insights into tumor biology.

## Materials and methods

### Mutation data

A total of 183,916 somatic point mutations found in the genomes of *M *= 21 different breast cancers [13], were subjected to analysis. Each mutation was mapped to one of *N*_*c *_= 96 trinucleotide mutation channels, defined by one of six possible unique base pair changes (C:G > A:T, C:G > G:C, C:G > T:A, T:A > A:T, T:A > C:G and T:A > G:C) and one of the sixteen different trinucleotide sequence contexts (as given by the bases immediately 5' and 3' to the mutated site in a pyrimidine context). As such, the combined observations were described by the matrix $X_{j}^{m}\left(j=1,\ldots ,N_{c},m=1,\ldots ,M\right)$ of channel- and tumor-specific mutation counts.

### Elementary processes of mutation

We assume that the observed mutational data can be explained by the action of *n *distinct elementary mutational processes, denoting the mutational signatures of the processes as {*μ_aj_*} (*a *= 1,...,*n, j *= 1,...,*N_c_*), where $\sum_{j=1}^{N_{c}}\mu_{aj}=1$. We denote the activity of process *a *in tumor *m *as $x_{a}^{m}$, representing the extent to which a process has been operative in a given tumor. It is important to note that a derivation of the inherent mutation rate of a process is not possible; the total activity of a process in a given cancer is the product of its bare mutation rate, the time for which it has been active and the proportion of the genome that was actually attacked. While this last contribution could be factored out with a spatially resolved analysis, the first two are inseparable without time-resolved sequencing of the tumor.

Given the above, we describe the result of a stochastic infusion of mutations into the genome using a Poisson distribution. Assuming the mutational processes to be mutually independent, the probability of observing the vector *X^m ^*of mutation counts in tumor *m *across channels *j *is given by:

(1) $$P\left(X^{m}|x^{m},\omega^{m},\mu \right)\equiv \prod_{j=1}^{N_{c}}\text{Pois }\left(X_{j}^{m}|\overset{n}{\underset{a=1}{\sum }}x_{a}^{m}\mu_{aj}\omega_{j}^{m}\right),$$

where $\omega_{j}^{m}$ is the opportunity for mutations in channel *j *to occur in tumor *m*. In the data set under study, copy number variation produces differences in the mutational opportunity between tumors; we have here factored in the available ploidy information [19] in the following way: each base pair can mutate in three different ways corresponding to three of the 96 mutational channels, depending on the immediate sequence context of the mutated base. The total mutational opportunity vector *ω^m ^*is the cumulative sum over all bases, where at each base three channels are increased not by one, but by the copy number of that base in genome *m*. In a variant of the method, which ignored mutational opportunity, the values $\omega_{j}^{m}$ were set to 1. We note that, of the parameters in equation 1, the activities *x *and the spectra *μ *are unknown. Maximum likelihood estimation of these values was accomplished using an EM algorithm.

### Finding the mutational spectra

The EM algorithm finds maximum likelihood (ML) estimates of the unknown shared model parameters (here the spectra *μ*) and the hidden data (here the activities *x*). After an initial guess, *μ*^(0)^, is made, two steps are repeated in an iterative fashion. In the first step, given the current best guess, *μ*^(*k*)^, and the observed data, *X*, an estimate is found for *x*. In the second step, this estimate, $\hat{x}$ of the hidden data is used to obtain the updated parameter estimate, *μ*^(*k *+ 1)^. These two steps are iterated until convergence to a (local) maximum of the data likelihood, *P*(*X*|*μ*), is achieved [17]. More formally, the routine proceeds as follows:

0. (Initialize) Choose an initial guess for *μ*, respecting the normalization $\sum_{j=1}^{N_{c}}\mu_{aj}=1$.

1. (E-step) Given the current estimate *μ*^(*k*)^, find maximum likelihood estimates for all the hidden activities:

(2) $${\hat{x}}^{m}\equiv \underset{x\in {\mathbb{R}}_{+}^{n}}{\text{argmax}}\;\;\text{log }P\left(X^{m}|x,\omega^{m},\mu^{\left(k\right)}\right)=\underset{x\in {\mathbb{R}}_{+}^{n}}{\text{argmin}}\overset{N_{c}}{\underset{j=1}{\sum }}\left[\overset{n}{\underset{a=1}{\sum }}x_{a}\mu_{aj}^{\left(k\right)}\omega_{j}^{m}-X_{j}^{m}\text{ log }\left(\overset{n}{\underset{a=1}{\sum }}x_{a}\mu_{aj}^{\left(k\right)}\omega_{j}^{m}\right)\right]$$

2. (M-step) Using above hidden data estimates, update the mutation spectra according to:

(3) $$\mu^{\left(k+1\right)}\equiv \underset{\mu \in {\mathbb{R}}^{n\;\times \;N_{c}}}{\text{argmax}}\;\;\overset{M}{\underset{m=1}{\sum }}\text{log }P\left(X^{m}|{\hat{x}}^{m},\omega^{m},\mu \right),\text{with}\;\text{constraint }\overset{N_{c}}{\underset{j=1}{\sum }}\mu_{aj}^{\left(k+1\right)}=1.$$

3. (Finish) Test for convergence. If needed, go back to the E-step and repeat.

This formulation of the EM routine follows from the more standard formulation [17] (a rigorous derivation is given in Additional file 1). The performance of the EM algorithm depends heavily on how quickly and accurately the two maximizations can be carried out. Here, approximate analytical solutions to both steps were used to significantly speed up the route to convergence (see Additional file 1); numerical optimization was used for the final steps of the iteration only.

### Calculating the total data likelihood

After convergence of the EM algorithm to a maximum likelihood estimate, $\hat{\mu }$, of the *n *spectra, the numerical value of the likelihood was calculated by integrating out the latent variables, *x*. To avoid the expense of numerical integration, this was performed using the saddle point approximation:

(4) $$\text{log}\;P\left(X|\hat{\mu }\right)=\overset{M}{\underset{m=1}{\sum }}\text{log }\int d^{n}x\;P\left(X^{m}|x,\omega^{m},\hat{\mu }\right)\approx \overset{M}{\underset{m=1}{\sum }}\left[\frac{n}{2}\text{log }2\pi -L\left({\hat{x}}^{m}\right)-\frac{1}{2}\text{log }\text{det }H\left(L\right)\left({\hat{x}}^{m}\right)\right]$$

where *L*(*x*) = -log *P*(*X*|*x, ω, μ*) is the conditional data log-likelihood (see equation 2), and *H *(*L*)(*x*) is its Hessian matrix of second derivatives.

### Finding the number of mutational processes

Comparing the data likelihoods obtained with different values of *n *indicated the most likely number of mutational processes. Increasing the value of *n *increases the number of model parameters available for fitting, and generally produces a better explanation for the data, *X*. This is reflected in an increased value of the likelihood, $P\left(X|\hat{\mu }\right)$. To avoid overfitting of the data, the BIC was used to correct for model complexity. The model with the largest value of BIC was ultimately selected [18,25]:

(5) $$\text{BIC}=2\text{ log }P\left(X|\hat{\mu }\right)-n\left(N_{c}-1\right)\text{ log }M.$$

### Estimating process activities and assigning mutations to processes

#### Global analysis

Completion of the EM algorithm provides maximum likelihood estimates, $\hat{\mu }$, of the mutational signatures of the *n *processes. Likewise, the E-step yields estimates, $\left\{{\hat{x}}_{a}^{m}\right\}$, of the process activities for each sample. Together, these can be used to estimate the number of mutations that was most likely contributed by each process to the set of all observed mutations: $X^{m}=\sum_{a=1}^{n}{\hat{X}}_{a}^{m}$. To improve the activity estimation, we generalize the simple maximum log-likelihood estimate of equation 2 to include prior information in the form of pseudocounts. As we show below, these pseudocounts are generally not uniform across channels. The activity estimates, $\left\{{\hat{x}}_{a}^{m}\right\}$, then have the status of a *maximum a posteriori*. Finally, the number of mutations, ${\hat{x}}_{a}^{m}$, in cancer *m *which were emitted in channel *j *by process *a *is then estimated as:

(6) $${\hat{x}}_{a,j}^{m,g}=\frac{\left|X^{m}\right|{\hat{x}}_{a}^{m,g}{\hat{\mu }}_{a,j}\omega_{j}^{m}}{\sum_{b=1}^{n}{\hat{x}}_{b}^{m,g}{\left(\hat{\mu }\omega^{m}\right)}_{b}},\text{where }\left|X^{m}\right|\equiv \overset{N_{c}}{\underset{j=1}{\sum }}X_{j}^{m},\;\text{and }{\left(\hat{\mu }\omega^{m}\right)}_{a}\equiv \overset{N_{c}}{\underset{j=1}{\sum }}\mu_{aj}\omega_{j}^{m}.$$

Here, the superindex *g *signifies a globally derived quantity. The assignment rule can be understood as follows: the fraction of observed mutations that were produced by a particular process must be proportional to its activity, its mutational spectrum and to the mutational opportunity in that channel. To each observation, *X^m^*, a total of *n *pseudocounts were added. These counts were assigned exactly in the above way, but without any bias due to the (as yet unknown) activities:

(7) $${\tilde{X}}_{aj}^{m}\equiv \frac{n{\hat{\mu }}_{aj}\omega_{j}^{m}}{\sum_{b=1}^{n}{\left(\hat{\mu }\omega^{m}\right)}_{b}}.$$

Thus, they represent an observation that is solely based on information known prior to the activity estimate. The pseudocounts enter the log-likelihood function (see equation 2) by an additional term:

(8) $$L\left(x;X^{m},\omega^{m},\mu \right)\to L\left(x;X^{m},\omega^{m},\mu \right)-\overset{N_{c}}{\underset{j=1}{\sum }}\overset{n}{\underset{a=1}{\sum }}{\tilde{X}}_{aj}^{m}\text{ log }\left(x_{a}\mu_{aj}\omega_{j}^{m}\right).$$

#### Local analysis

With exactly the same logic as above, we derived estimates of the local activity of each process within each cancer, dividing every genome into non-overlapping windows of length 1 Mb. However, we could now use the previously calculated global activities, $\left\{{\hat{x}}_{a}^{m,g}\right\}$, as a more informed prior for the inference of process activities within each megabase window, *l*:

(9) $${\tilde{X}}_{aj}^{m,l}=\frac{n\;{\hat{x}}_{a}^{m,g}{\hat{\;\mu }}_{aj}\omega_{j}^{m,l}}{{\sum_{b=1}^{n}{\hat{x}}_{a}^{m,g}\left(\hat{\mu }\omega^{m,l}\right)}_{b}}\overset{\text{E - step}\;\text{with}\;\text{eq}.\;8}{\underset{\text{using}\;X^{m,l},\omega^{m,l}}{\to }}{\hat{x}}_{a}^{m,l}.$$

These locally inferred activities, ${\hat{x}}_{a}^{m,l}$, were used to assign the mutations observed in each window to the *n *different processes, in the manner of equation 6. We also performed a consistency check of the local mutation assignments, measuring for each process independently whether the total effect of their local activities across all windows was consistent with the global activity provided by the global EM result; in more formal notation, we compared $\sum_{l=1}^{N_{b}}{\hat{X}}_{a}^{m,l}$ with ${\hat{X}}_{a}^{m,g}$ (where *N_b _*is the total number of bins). While there exists no *a priori *reason to expect a perfect agreement between these values, using the informed prior, the consistency was very high (see Additional file 1). A large deviation between values would signify that the local estimates are incompatible with the global estimate, suggesting a general discrepancy between the observed counts and the generative model used to assign them to processes.

### Identification of chromatin state

Chromatin states across the genome were identified using data from the ENCODE project describing a cell line of normal HMECs [20]. Sites within the genome were classified into seven broad chromatin states: promoter, enhancer, insulator, transcription, repressed, heterochromatin and repetitive regions [21].

### Comparison with an earlier approach

Both similarities and critical differences exist between our probabilistic approach and the non-negative matrix factorization (NMF) [11,12] employed in a previous analysis of the breast cancer data [13], and further documented elsewhere [26]. In general, NMF is a tool for a very particular task, finding the matrices, *x *and *μ*, that minimize some matrix norm, ||*X *- *xμ*||, without explicit reference to any probabilistic model. The iterative NMF algorithm can, however, be interpreted as an application of the EM paradigm [27], where different choices of matrix norms correspond to different assumptions about the underlying probabilistic model for the observed data, *X *(given *x *and *μ*). The discrete nature of mutation counts suggests a Poisson generative model, which, in turn, corresponds to a very particular matrix norm (related to the Kullback-Leibler divergence) that is minimized by NMF [27]. Differences between our approach and previous work fall into two categories. Firstly, our work addresses new concepts and applications in identifying mutational processes. The sequence-specific opportunity for mutations to occur, the inference of process activities in local regions of the genome, identification of *kataegis *events with a specific mutational process and the assessment of the impact of chromatin state, were not previously considered in this context. Some of these concepts could potentially be incorporated into an NMF-based framework, for example by scaling the numbers of observed mutations to account for tumor-specific mutational opportunities. Secondly, our method is based on a distinctly different mathematical formulation. Our belief is that the concrete probabilistic nature of the method presented here is an advantage for studying mutational processes, which are themselves inherently stochastic. For smaller genomic segments the sequence contexts become highly variable and the actual mutation counts become small necessitating a probabilistic treatment. Critically, our method allows for an assessment of statistical significance - even a small number of mutations can be of great interest provided they are of the right type in the right genomic context. More generally, use of a likelihood framework allows for the application of multiple statistical techniques. Monte Carlo methods such as MCMC or simulated annealing can be applied to find robust point estimates of the parameters, *μ*, and informative estimates of their errors. The ability to call upon existing theory was also of help in identifying the number of mutational processes; the BIC method applied in this study [18,25] provided a valuable criterion in selecting the correct number of mutational processes for our system.

## Data access

The current version of EMu is provided as Additional file 2. The most up-to-date version of EMu is available at [28] together with the localization results (see Figure 3) for all 21 cancers in computable document format (cdf) (viewable with Wolfram's free CDF Player [29]).

## Abbreviations

BIC: Bayesian information criterion; cdf: computable document format; EM: expectation-maximization; ENCODE: Encyclopedia of DNA Elements; HMEC: human mammary epithelial cells; MCMC: Markov chain Monte Carlo; ML: maximum likelihood; NMF: non-negative matrix factorization

## Competing interests

The authors declare that they have no competing interests.

## Authors' contributions

AF designed the study, carried out the calculations, wrote code, analyzed data, interpreted results and wrote the manuscript. CI participated in the design of the study and the interpretation of results, and wrote the manuscript. PC participated in the design of the study and the interpretation of results. VM designed the study, supervised the study, interpreted results and wrote the manuscript. All authors read and approved the final manuscript.

## Supplementary Material

Additional file 1 **Supporting information**. Explicit calculations and implementation for the methods presented in the main text as well as additional analysis of the breast cancer data. Click here for file

Additional file 2 **Software**. For archival purposes, the current version of EMu is included as an Additional File. However, we recommend you download the software from http://www.sanger.ac.uk/resources/software/emu/, in case a more up-to-date version has been released. Click here for file
