# Supplementary material for: EMu: probabilistic inference of mutational processes and their localization in the cancer genome
Source: Genome Biol. 2013 Apr 29;14(4):R39. doi: 10.1186/gb-2013-14-4-r39 (PMC3663107; doi:10.1186/gb-2013-14-4-r39)
Supplement: Additional file 1 — Supporting information. Explicit calculations and implementation for the methods presented in the main text as well as additional analysis of the breast cancer data. [file gb-2013-14-4-r39-S1.PDF]

Supporting Text: Probabilistic Inference of Mutational Processes and their Localisation in the Cancer Genome

Andrej Fischer[1], Christopher J.R. Illingworth[1], Peter J. Campbell[1] and Ville Mustonen*[1]

[1]Wellcome Trust Sanger Institute, Wellcome Trust Genome Campus, CB10 1SA, Hinxton, Cambridge, UK

Email: Andrej Fischer - af7@sanger.ac.uk; Christopher J.R. Illingworth - ci3@sanger.ac.uk; Peter J. Campbell - pc8@sanger.ac.uk; Ville Mustonen*- vm5@sanger.ac.uk;

*Corresponding author

1 Derivation of the EM algorithm for mutation spectra

1.1 The generative model

The EM algorithm presented in this work is an application of the method first described in [1]. The starting point of the derivation is the conditional data likelihood, i.e. the probability of observed data (the mutation counts X), conditional on both hidden data (the process activities x) and the model parameters (the mutation spectra μ). The mutations are produced jointly by n independent Poisson processes, such that the Poisson rate for the joint process is the sum of the individual rates.

$$P\left(X^m \mid x^m, \omega^m, \mu\right) = \prod_{j=1}^{N_c} \sum_{c_{j1}..c_{jn}} \delta\left(X_j^m - \sum_{a=1}^{n} c_{ja}\right) \prod_{a=1}^{n} \mathrm{Pois}(c_{ja} \mid x_a^m\ \mu_{aj}\ \omega_j^m) \tag{S1}$$

$$= \prod_{j=1}^{N_c} \mathrm{Pois}\left(X_j^m \middle| \sum_{a=1}^{n} x_a^m\ \mu_{aj}\ \omega_j^m\right) \equiv \exp\left[-L\left(x^m; X^m, \omega^m, \mu\right)\right] \tag{S2}$$

$$L\left(x^m; X^m, \omega^m, \mu\right) = \sum_{j=1}^{N_c}\left[\sum_{a=1}^{n} x_a^m\ \mu_{aj}\ \omega_j^m - X_j^m\ \log\left(\sum_{a=1}^{n} x_a^m\ \mu_{aj}\ \omega_j^m\right) + \log\left(X_j^m!\right)\right] \tag{S3}$$

The last line is the negative log-likelihood L expressed as a function of the hidden data x. We observe that the hidden data and the parameters are not uniquely determined by the observed data, since only the combinations $x_a^m \mu_{aj}$ enter above probability. These n degrees of freedom are eliminated by the n constraints

$$\forall\ a = 1 \ldots n: \quad \sum_{j=1}^{N_c} \mu_{aj} = 1. \tag{S4}$$

In this sense, we call the μ the mutational *spectra* of the processes. Note, that the total rate for a process $x_a^m \mu_{aj}$ must be proportional to the true underlying bare *mutation rate* μ_{aj}^0 of that process (measured in

some appropriate time units), the total duration τ_a^m that this process has been active in that tumour and eventually the fraction p_a^m of the cancer genome that is thus affected:

$$\forall j, a, m : \quad x_a^m \mu_{aj} \propto \mu_{aj}^0 \ \tau_a^m \ p_a^m . \tag{S5}$$

There is no way that the EM analysis can learn the τ's and the p's independently. Without some additional molecular clock to fix the time scales, we can not even differentiate between high *activities* $x_a^m = \tau_a^m p_a^m$ (exposition duration times prevalence) and high absolute mutation rates μ_{aj}^0.

1.2 The EM-Q-function

The EM-Q-function is the central quantity for the parameter estimation [1]. It involves integrals over the hidden data. The exponential form of the probability above suggests that these integrals can be subjected to a saddle point approximation. For completeness, we also formally include any prior information I_0.

$$Q\left(\mu^{(k)} \,\middle|\, \mu\right) = \sum_{m=1}^{M} \int \mathrm{d}^n x \ P\left(x \mid X^m, \omega^m, \mu^{(k)}, I_0\right) \ \log \ P\left(x, X^m \mid \omega^m, \mu, I_0\right) \tag{S6}$$

$$= \sum_{m=1}^{M} \ \int \mathrm{d}^n x \ \frac{P\left(X^m \mid x, \omega^m, \mu^{(k)}, I_0\right) \ P\left(x \mid I_0\right)}{P\left(X^m \mid \omega^m, \mu^{(k)}, I_0\right)} \ \log \ P\left(x, X^m \mid \omega^m, \mu, I_0\right) \tag{S7}$$

$$= \sum_{m=1}^{M} \frac{\int \mathrm{d}^n x \ e^{-L(x)} \ P\left(x \mid I_0\right) \ \log \ \left[P\left(X^m \mid x, \omega^m, \mu, I_0\right) \ P\left(x \mid I_0\right)\right]}{\int \mathrm{d}^n x \ e^{-L(x)} \ P\left(x \mid I_0\right)} \tag{S8}$$

$$\approx \sum_{m=1}^{M} \sum_{j=1}^{N_c} \left[X_j^m \log \left(\sum_{a=1}^{n} \hat{x}_a^m \ \mu_{aj} \ \omega_j^m \right) - \sum_{a=1}^{n} \hat{x}_a^m \ \mu_{aj} \ \omega_j^m \right] + \sum_{m=1}^{M} \log P\left(\hat{x}^m \mid I_0\right) . \tag{S9}$$

As is often the case in EM calculations, the saddle point approximation proofs quite useful since it is applied in both the numerator and the denominator, such that most terms cancel (e.g. the determinant of the Hessian matrix of second derivatives). All the saddle points $\hat{x}^m$ depend on the current best estimate of the model parameters.

$$\forall \ m = 1 \ldots M : \quad \hat{x}^m \ = \ \operatorname*{argmin}_{x \in \mathbb{R}^n_{\geq 0}} \ L\left(x; \ X^m, \omega^m, \mu^{(k)}, I_0\right) \tag{S10}$$

In the main text, we have argued that we can incorporate prior information I_0 indirectly via pseudo-counts. The last term in above Q-function is then changed accordingly (see eq. 8).

1.2.1 Approximative calculation of the saddle points

For each of the M samples, the saddle point $\hat{x}^m$ must be calculated according to eq. S10. While this can be done numerically, an approximation to the saddle point can be found by solving two auxiliary problems:

$$1.\ \ \hat{y}^m = \underset{y \in \mathbb{R}_{\geq 0}^{N_c}}{\operatorname{argmin}} \sum_{j=1}^{N_c} \left(y_j - X_j^m \ \log y_j \right) = X^m \tag{S11}$$

$$2.\ \ \hat{x}^m = \underset{x \in \mathbb{R}_{\geq 0}^{n}}{\operatorname{argmin}} \left\| X^m - d\left(\omega^m\right) \mu^T x \right\| = \left(\mu \ d^2(\omega^m) \ \mu^T \right)^{-1} \mu \ d(\omega^m) \ X^m \tag{S12}$$

$$\text{with} \quad d_{ij}(\omega^m) = \omega_j^m \ \delta_{ij}, \quad i, j = 1 \ldots N_c \tag{S13}$$

The second step is a standard linear least square minimisation problem and is solved with the normal equation. This approximation will *not* yield the exact saddle point and thus lead to systematic errors in the EM iteration, unless the parameter update that is based on this approximation still increases the EM-Q-function. This would be sufficient for the EM algorithm to converge to a (local) maximum of the likelihood.

1.3 The parameter update

With the EM-Q-function at hand, we can proceed to find an update to the mutation spectra μ. The maximisation of the Q-function is actually a similar problem to what we had above in the E-step.

$$\mu^{(k+1)} = \underset{\mu \in \mathbb{R}_{\geq 0}^{n \times N_c}}{\operatorname{argmax}} \ Q\left(\mu^{(k)} \,\middle|\, \mu \right) \tag{S14}$$

$$Q\left(\mu^{(k)} \,\middle|\, \mu \right) = \sum_{m=1}^{M} \sum_{j=1}^{N_c} \left[X_j^m \log \left(\sum_{a=1}^{n} \hat{x}_a^m \mu_{aj} \omega_j^m \right) - \sum_{a=1}^{n} \hat{x}_a^m \mu_{aj} \omega_j^m \right] \tag{S15}$$

This task factorizes over the N_c channels and we can give the same approximative solution as before:

$$\mu_j^{(k+1)} \approx \underset{\mu_j \in \mathbb{R}_{\geq 0}^{n}}{\operatorname{argmin}} \left\| X_j - d(\omega_j) \ \hat{x} \ \mu_j \right\| = \left(\hat{x}^T \ d^2(\omega_j) \ \hat{x} \right)^{-1} \ \hat{x}^T \ d(\omega_j) \ X_j \tag{S16}$$

$$\text{with} \quad \left(X_j\right)_m = X_j^m, \quad d_{mk}(\omega_j) = \omega_j^m \delta_{mk}, \quad m, k = 1 \ldots M \tag{S17}$$

The two equations S12 and S16 can be used in the initial phase of the EM iteration to reach the vicinity of the true maximum of the data likelihood. Then their exact numerical variants are applied, of which much fewer evaluations are now needed until convergence.

1.4 The data likelihood $P\,(X \mid \mu, \omega)$

To evaluate the data log-likelihood for given parameters, we employ again the saddle point approximation:

$$\log\ P\,(X \mid \mu, \omega) = \sum_{m=1}^{M} \log \int \mathrm{d}^n x\ P\,(X^m \mid x,\, \mu, \omega^m) \tag{S18}$$

$$= \sum_{m=1}^{M} \log \int \mathrm{d}^n x\ e^{-L(x;\ X^m,\, \mu, \omega^m)} \tag{S19}$$

$$\approx \sum_{m=1}^{M} \left[\frac{n}{2} \log 2\pi - L\,(\hat{x}^m) - \frac{1}{2} \log\det\ \mathrm{H}(L)\,(\hat{x}^m) \right] \tag{S20}$$

Note that there is no cancellation of terms as before. Also note, that the saddle point approximation is only possible, if the Hessian matrix $\mathrm{H}(L)(x)$ of second partial derivatives is positive-definite. A necessary condition is that at least n entries of X^m must be positive. This follows directly from

$$\mathrm{H}_{ab}(L)\,(\hat{x}^m) = \sum_{j=1}^{N_c} \frac{X_j^m\ \mu_{aj}\ \mu_{bj}}{\left(\sum_{c=1}^{n}\ \hat{x}_c^m\ \mu_{cj}\right)^2}. \tag{S21}$$

Explicit inclusion of pseudo-counts alleviates this particular pathology. For this reason, we have included pseudo-counts as in eq. 7 also in the global EM analysis.

1.5 Error estimates for the mutation spectra

In a sense, the above data-likelihood is the central quantity for the inference of the mutation spectra. Without informative prior information on μ, it is proportional to the posterior distribution $P\,(\mu \mid X, \omega)$, of which the EM algorithm effectively finds a local maximum. At that maximum, we can approximate this distribution again by a Gaussian and use that to quantify the uncertainty of the inference.

$$F(\mu; X, \omega) \equiv \log\ \ P\,(X \mid \mu, \omega) \quad \Rightarrow \quad \mathrm{var}(\mu_{aj}) \approx -\mathrm{H}^{-1}_{aj,aj}(F)(\hat{\mu}) \tag{S22}$$

$$\text{with}\quad \mathrm{H}_{aj,bk}(F)(\hat{\mu}) = \left(\partial_{\mu_{aj}} \partial_{\mu_{bk}} F\right)(\hat{\mu}; X, \omega) \qquad (i, j = 1 \ldots N_c,\ a, b = 1 \ldots n) \tag{S23}$$

$$\approx -\sum_{m=1}^{M} \left(\partial_{\mu_{aj}} \partial_{\mu_{bk}} L\right)(\hat{x}^m; X^m, \hat{\mu}, \omega^m) \tag{S24}$$

$$= -\sum_{m=1}^{M} \delta_{jk} \left(\frac{\hat{x}_a^m\ \hat{x}_b^m\ X_j^m}{\left(\sum_{c=1}^{n} \hat{x}_c^m\ \hat{\mu}_{cj}\right)^2} + \delta_{ab}\, \frac{\tilde{X}_{aj}^m}{\hat{\mu}_{aj}^2} \right). \tag{S25}$$

where the integrals over x are evaluated in saddle point approximation, as described above. To verify these estimates, we also probed the posterior distribution directly with a Markov chain Monte-Carlo simulation (MCMC) [2–4]. The error estimates derived by both methods were highly consistent (see Figure S1).

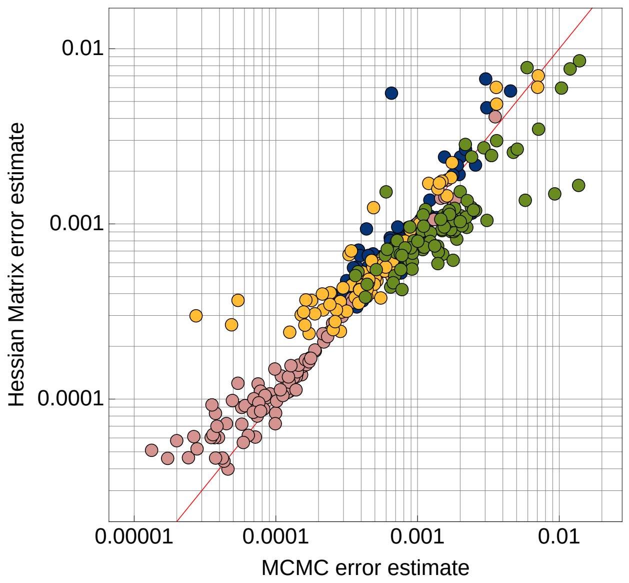

Figure S1: Comparison of the error estimates for the four mutational spectra found in the 21 breast cancers as shown in Figure 1. Each dot is the error estimate for the mutation probability in one of the 96 channels, derived from 10^5 MCMC steps (horizontal axis) and using the approximation in eq. S22.

2 Implementation of EMud

EMu, the EM-analysis of mutation data, is implemented as a C++ application that relies on the GNU scientific library version 15 [2]. As input, the program takes the matrix of mutation counts X and the corresponding matrix of mutational opportunities ω. As a first step, the number of independent spectra supported by the data is found by applying a quick but coarse variant of EM (with few iterations involving numerical minimisation, but 10 random starting points) for successive values of n. As soon as a maximum in BIC is reached, the program then carries out a refined version of EM for this number of processes. The capability to append a MCMC sampling of $P(\mu \mid X, \omega)$ after the EM convergence is built into the software.

3 Simulated mutation data sets

We simulated mutation data sets to investigate the performance of the EM method with increasing number of active processes and number of available samples. In realistic scenarios, the EM is either applied to data from a single cancer type with few mutational processes or to large data sets encompassing different cancer types, all with their own unique set of signatures. We simulated data sets for the case of one, two and three cancer types with five mutational processes each. Each simulated tumour was assigned to a single cancer type (one of up to three). Thus, only ever five different processes contributed the mutations. The

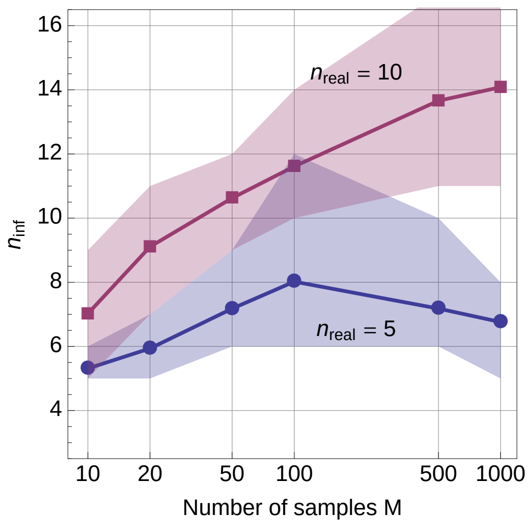

Figure S2: The number of inferred spectra as a function of sample size for $n_{\mathrm{real}} = 5$ and 10, as given by BIC and ignoring mutational opportunity. Shown is the median over 50 replicates for each combination of n and M (line), as well as the minimum and maximum value (shaded area).

individual process specific mutational signatures were drawn from a uniform distribution over 96 mutation channels. The process activities x^m for each tumour were then drawn from a uniform distribution over the 5-dimensional simplex and multiplied with a variate from an exponential distribution with mean 10^{-4}. The mutational opportunity was derived from the human reference genome sequence (release 37) and kept fixed for all simulated tumours (ignoring tumour specific ploidy changes). The actual mutation counts were finally generated using the generative model (see eq. 1 in the main text) and typically ranged between 10 and 10^4 mutations genome wide. The time measurements in Figure 1 show the run time of the inference program on a typical processor in the Sanger Institute compute cluster (2x3.0 Ghz quad core Intel E5450) using openMP parallelisation with four threads.

The importance of mutational opportunity in the inference

To demonstrate that the information about the mutational opportunity ω is indispensable for the reconstruction of the simulated data sets, we performed above analysis also without its consideration (setting all $\omega_j^m = 1$). Using the BIC, the number of true processes was consistently over-estimated (see Figure S2). This is due to fact that the heterogenous opportunity spectra introduce additional variation in the mutation count data that the EM algorithm then tries to capture with additional mutation spectra.

4 Analysis of mutations in 21 breast cancers

4.1 The impact of mutational opportunity and PD4120a on the EM result

As was mentioned in the main text, the mutational opportunity ω entered the EM analysis as fixed tumour specific parameters. In principle, the EM can be carried out ignoring this information completely by setting all $\omega_j^m = 1$ and fixing the number of spectra. The result is then comparable to the analysis carried out in [5], where non-negative matrix factorisation (NMF) was employed for $n = 5$. The spectra found by our EM, with $\omega = 1$ and n forced to five, can be seen in Figure S3, where the spectra of [5] are essentially fully recovered. It is important to note that NMF itself does not provide an intrinsic model selection criterion. With the probabilistic approach of this paper the BIC is a meaningful criterion that makes, however, only sense under explicit consideration of the mutational opportunity (see above).

4.2 Index of dispersion

Are the mutations per process randomly distributed along the genome in a Poisson-like fashion, or do they cluster in specific sub regions? To answer this question, we must take into account the highly heterogenous spectrum-opportunity overlap, which would – even under the null hypothesis of constant activity – result in apparent local clustering. One measure that captures clustering is the index of dispersion, i.e. the mean of counts per bin divided by the variance. It takes a value of one for standard Poisson processes. However, for our analysis the value is changed even under the random null-model due to the heterogeneity in mutational opportunity (N_b is the number of 1 Mb bins per genome).

$$H_0: \quad X_a^{m,l} \sim \text{Pois}\left(\hat{x}_a^{m,g}\left(\mu\,\omega^{m,l}\right)_a\right) \tag{S26}$$

$$m_1 \equiv \frac{1}{N_b}\sum_{l=1}^{N_b} X_a^{m,l} \;\Rightarrow\; \langle m_1\rangle_0 = \hat{x}_a^{m,g}\frac{1}{N_b}\sum_{l=1}^{N_b}\left(\mu\,\omega^{m,l}\right)_a = \hat{x}_a^{m,g}\;\langle\left(\mu\,\omega^{m,l}\right)_a\rangle \tag{S27}$$

$$m_2 \equiv \frac{1}{N_b}\sum_{l=1}^{N_b}\left(X_a^{m,l}\right)^2 \;\Rightarrow\; \langle m_2\rangle_0 = \hat{x}_a^{m,g}\;\langle\left(\mu\,\omega^{m,l}\right)_a\rangle + \left(\hat{x}_a^{m,g}\right)^2\;\langle\left(\mu\,\omega^{m,l}\right)_a^2\rangle \tag{S28}$$

$$\frac{\langle m_2\rangle_0 - \langle m_1\rangle_0^2}{\langle m_1\rangle_0} = 1 + \hat{x}_a^{m,g}\;\frac{\langle\left(\mu\,\omega^{m,l}\right)_a^2\rangle - \langle\left(\mu\,\omega^{m,l}\right)_a\rangle^2}{\langle\left(\mu\,\omega^{m,l}\right)_a\rangle} \tag{S29}$$

To test for clustering of the mutations, we calculate the observables m_1 and m_2 and divide the resulting index of dispersion by the null-expectation, i.e. the right hand side of the last equation above.

4.3 Correlation of process mutations with histone modifications

Histone modifications – methylations and acetylations – are marks for chromatin accessibility and thus relate to the functional state of a genomic region [6]. Across several cancer types, the distribution of mutations

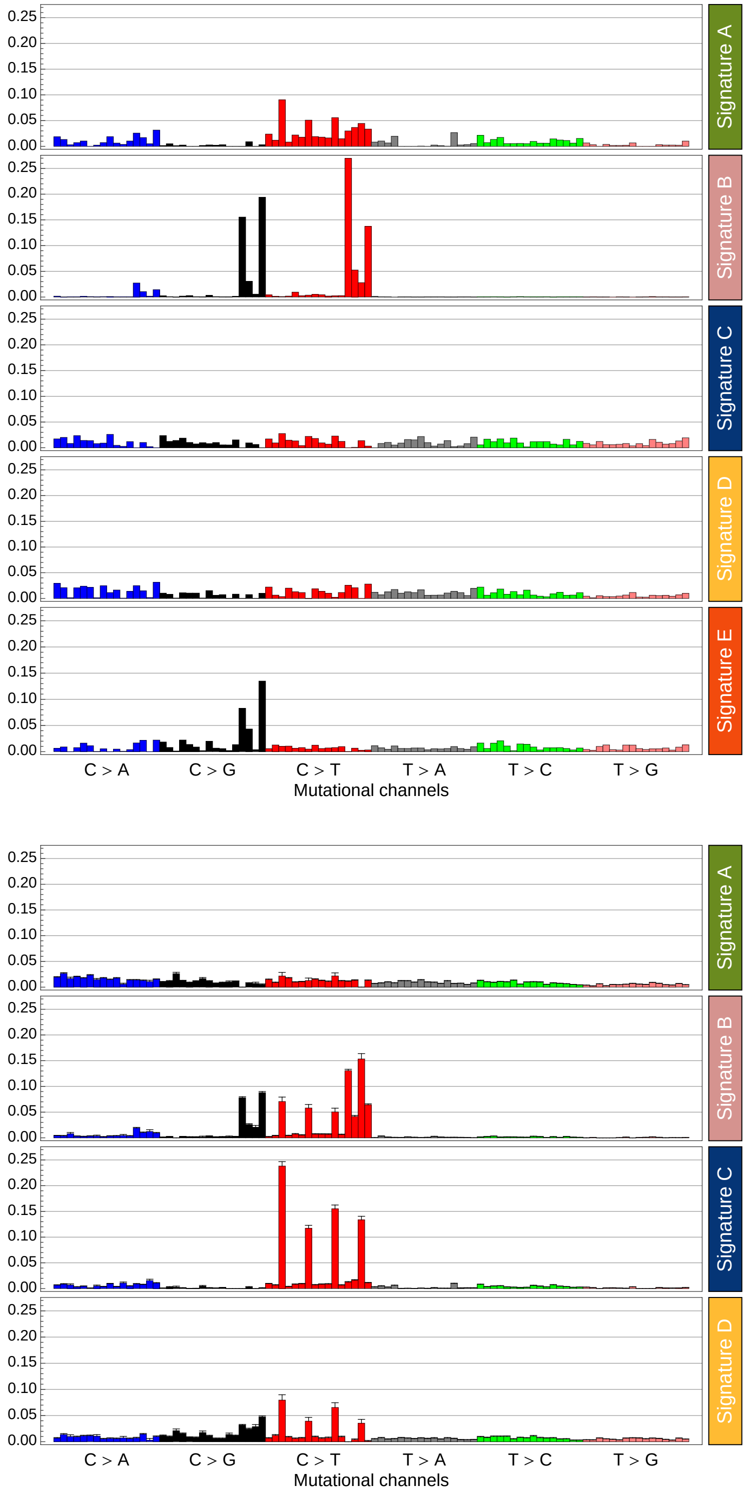

Figure S3: Top: The elementary signatures found by the EM analysis for $n = 5$ and ignoring the mutational opportunity information. Compare these spectra to the ones inferred in [5] (Figure 2 therein). Bottom: The signatures found by the EM analysis excluding the sample PD4120a. Process A, C and D are almost unchanged, but process B displays some admixture from the other two for C>T mutations in XpCpG contexts.

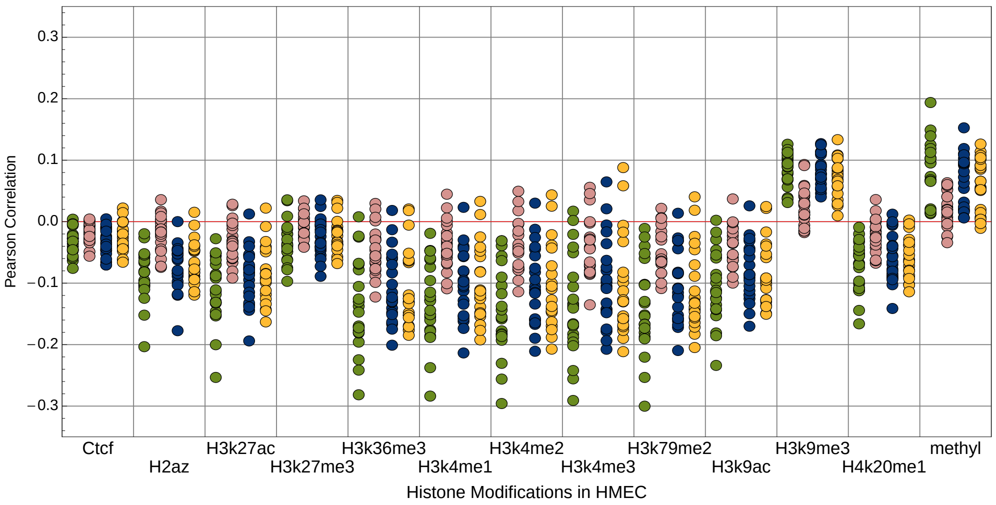

Figure S4: Correlation coefficient between the genome-wide distribution of various histone modification marks in a human mammary epithelial cell line (HMEC) and the process-specific mutations in breast cancer (on a mega-base scale, colours as in main Figure 2).

in the cancer genome was recently shown to be sensitive to some histone modifications, markedly H3k9me3 which is associated with heterochromatin [7]. For the breast cancer data at hand, we analysed the histone modifications for a normal breast tissue cell lines (HMEC, i.e. human mammary epithelial cells) available via the ENCODE database [8–10]. In Figure S4, we show the correlation coefficient between the distribution of histone modification density and the process specific distribution of cancer mutations (on a mega-base scale, correlations per sample).

Of the histone modifications available for breast related cell lines, H3k9me3 is the only one with sizeable positive correlations, confirming the finding in [7], albeit with lower values. All other marks show negative correlations, which are strongest for process A, C and D. Additionally, we correlated the mutations to the distribution of the fraction of methylated CpG sites per mega-base [9, 10]. Bins with a large fraction of methylated CpG tend to have more mutations of process A, C and D. This is consistent with a lack of repair of spontaneously deaminated 5-methylcytosines in a CpG context.

4.4 Significance of chromatin state activity variation

We compared the distribution of mutations in the cancer genomes with the functional segmentation into chromatin states as given by ENCODE [8–10]. To assess whether the individual mutational processes are sensitive to the functional state, we compared for each tumour m and process a and each chromatin state s two competing models: either the activity x_a^m is the same in the whole genome or there are two different

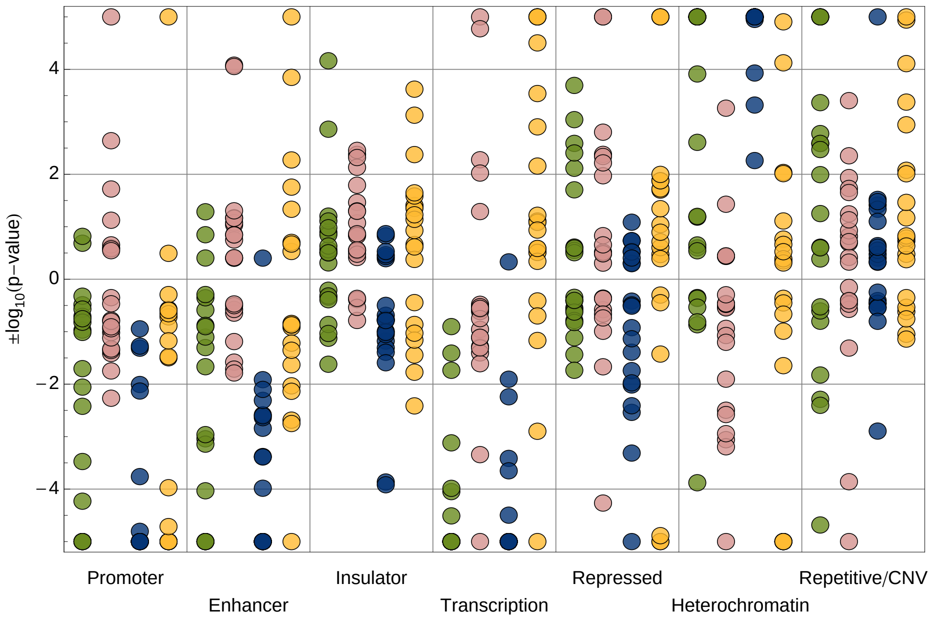

Figure S5: The significance level for the hypothesis that the activity of a process is sensitive to chromatin state (for each combination of chromatin state, mutational process and tumour; same process colours as in the main text). The p-values are capped at 10^{-5} and the sign indicates the direction of the bias. In almost all tumours, process C (dark blue, with a bias for C>T mutations at CpG sites) is almost surely sensitive to the "heterochromatin", "promoter" and "transcription" state of the genome.

activities, $x^m_{a,s}$ in state s and $x^m_{a,\bar{s}}$ in the genomic complement $\bar{s}$. Using the first as a null-hypothesis, we calculated p-values under the generative Poisson model for each combination of chromatin state, mutational process and tumour. This is exactly the same logic as for the localisation analysis based on Mb windows, where the local p-value expresses our surprise to see more (or less) mutations in a genomic region when we assume the null-model of uniform activity to hold. The results can be seen in Figure S5. The most consistent and strongest support for chromatin state sensitivity is seen for process C in "heterochromatin", "promoter" and "transcription" regions. The effect size of the change in activity can be seen in the main text (Figure 4).

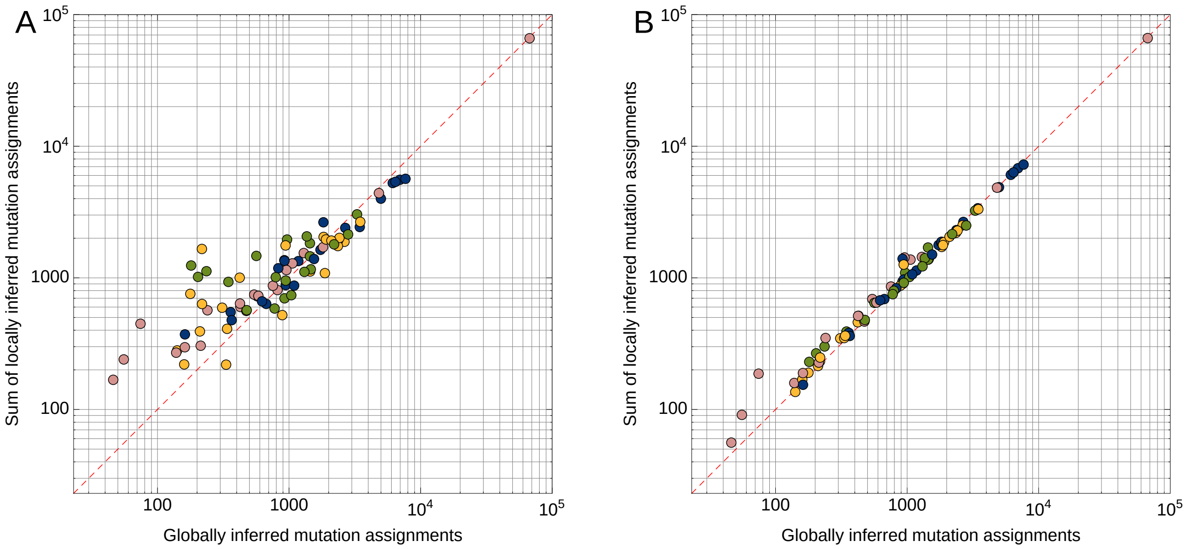

Figure S6: Consistency check: do the locally assigned mutations per process add up to the global assignment? Each dot represents one process in one sample. Inferring the activities for each mega-base bin independently leads to deviations between the two levels (left). The use of the globally inferred activity information as a prior for the local activity estimates increases the consistency between the two levels (right).

References

1. Dempster AP, Laird NM, Rubin DB: **Maximum likelihood from incomplete data via the em algorithm**. *Journal of the Royal Statistical Society: Series B* 1977, **39**:1–38.
2. Galassi M, et al.: *GNU Scientific Library Reference Manual (3rd edition)*. Network Theory Ltd., 3 edition 2009, [http://www.gnu.org/software/gsl/]. [ISBN 0954612078].
3. Roberts GO, Gelman A, Gilks WR: **Weak convergence and optimal scaling of random walk Metropolis algorithms**. *The Annals of Applied Probability* 1997, **7**:110–120.
4. Betancourt M: **Cruising the simplex: Hamiltonian Monte Carlo and the Dirichlet distribution** 2010, [http://arxiv.org/abs/1010.3436].
5. Nik-Zainal S, Alexandrov L, Wedge D, Van Loo P, et al.: **Mutational Processes Molding the Genomes of 21 Breast Cancers**. *Cell* 2012, **149**:979–993.
6. Barski A, Cuddapah S, Cui K, Roh TY, Schones DE, Wang Z, Wei G, Chepelev I, Zhao K: **High-Resolution Profiling of Histone Methylations in the Human Genome**. *Cell* 2007, **129**(4):823–837.
7. Schuster-Böckler B, Lehner B: **Chromatin organization is a major influence on regional mutation rates in human cancer cells**. *Nature* 2012, **488**:504–507.
8. Ernst J, Kheradpour P, Mikkelsen TS, Shoresh N, Ward LD, Epstein CB, Zhang X, Wang L, Issner R, Coyne M, Ku M, Durham T, Kellis M, Bernstein BE: **Mapping and analysis of chromatin state dynamics in nine human cell types**. *Nature* 2011, **473**(7345):43–49.
9. ENCODE Project Consortium, Myers R, Stamatoyannopoulos J, Snyder M, Dunham I, Hardison R, Bernstein B, Gingeras T, Kent W, Birney E, et al.: **A User's Guide to the Encyclopedia of DNA Elements (ENCODE)**. *PLoS Biology* 2011, **9**(4).
10. ENCODE Project Consortium, Bernstein BE, Birney E, Dunham I, Green ED, Gunter C, Snyder M: **An integrated encyclopedia of DNA elements in the human genome.** *Nature* 2012, **489**(7414):57–74.
